# The effect of calcineurin inhibitors on anthropometric measurements in kidney transplant recipients

**DOI:** 10.1186/s12882-022-03004-1

**Published:** 2022-11-19

**Authors:** Emel Isiktas Sayilar, Alparslan Ersoy, Canan Ersoy, Aysegul Oruc, Yavuz Ayar, Deniz Sigirli

**Affiliations:** 1 Departments of Nephrology, Uludag University Faculty of Medicine, Bursa, Turkey; 2 Present address: Department of Nephrology, Ufuk University School of Medicine Dr. Ridvan Ege Hospital, Mevlana Blv. No: 86-88, Balgat, Ankara, 06830 Turkey; 3 Endocrinology and Metabolism, Uludag University Faculty of Medicine, Bursa, Turkey; 4 Biostatistics, Uludag University Faculty of Medicine, Bursa, Turkey

**Keywords:** Kidney transplant recipients, Anthropometrics, Immunosuppression, Calcineurin inhibitors

## Abstract

**Background:**

This study was designed to investigate the effect of calcineurin inhibitors (CNIs), cyclosporine (CsA), and tacrolimus (Tac) on anthropometrics in kidney transplant recipients.

**Methods:**

111 of 128 adult kidney transplant recipients who received post-transplant CNIs were included in this retrospective study. Anthropometrics were recorded in the pre-transplant and post-transplant 4-year follow-up periods (1^st^, 3^rd^, 6^th^, 12^th^, 24^th^, 36^th^ and 48^th^ months).

**Results:**

Compared to pre-transplant values, significant increases in body weight and body mass index (between 3^rd^ and 48^th^ months), waist and hip circumferences (between 1^st^ and 48^th^ months), waist-to-hip ratio (between 1^st^ and 3^rd^ or 6^th^ months) and neck circumference (between 1^st^ and 12^th^ or 24^th^ months) were observed in both CsA and Tac groups. A significant increase was noted in post-transplant body fat percentage values for the 3^rd^ to 24^th^ months in the CsA group, whereas for the 24^th^ to 48^th^ months in both CsA and Tac groups. Hip circumferences percentage changes from the pre-transplant period to the 1^st^, 12^th^ and 24^th^ months were significantly higher in CsA than in the Tac group. At each time point, there was no significant difference in percentage changes for other anthropometric parameters between the CsA and Tac groups. De novo diabetes mellitus developed in 8.3% of the CsA group and 19.1% of the Tac group.

**Conclusions:**

After a successful kidney transplant, anthropometric measurements increase in most recipients. Although the effect of calcineurin inhibitor type on weight gain is unclear, a regression analysis showed that CNI type was not a risk factor for the development of obesity in the 48^th^ month. However, it is helpful to be cautious about its dyslipidemic effect in patients using CsA and the potential hazards of using Tac in patients with a diabetic predisposition.

## Introduction

Transplantation has become a commonly preferred renal replacement therapy option worldwide, offering improved survival, a better quality of life and lower treatment costs than dialysis [1, 2]. Calcineurin inhibitors (CNIs), including cyclosporine A (CsA) and tacrolimus (Tac), are the most potent immunosuppressive drugs, considered an important part of post-transplant immunosuppression therapy to prevent the rejection of transplanted kidneys [3, 4].

Weight gain and obesity in transplant patients show similar trends to those in the general population with a rising prevalence, affecting approximately 60% of patients within six years of transplantation [5, 6]. This seems notable given that obesity is considered a severe risk factor for graft function loss in the late post-transplant period and its association with proteinuria by causing hyperfiltration [7–9]. Some immunosuppressive drugs have been suggested to be associated with weight gain and obesity after kidney transplantation [10]. The prevalence of weight gain in the first year of transplantation is about 20%. Further evidence suggests that body composition may affect post-transplant prognosis [11, 12]. Abdominal obesity is evaluated by waist and hip circumference (HC) measurements, while the waist-to-hip ratio (WHR) is a valuable marker of central obesity and visceral fat. Recent studies suggest that body mass index (BMI) is not a good predictor of obesity, and that waist circumference (WaC) may be a better parameter [13–15]. The present study aimed to investigate the impact of post-transplant Tac and CsA treatments on the anthropometric parameters in kidney transplant recipients.

## Materials and methods

### Study population

This retrospective study included patients who had a successful kidney transplant from a living or deceased donor between May 2010 and December 2013. The study population consisted of 128 consecutive adult patients treated with CNI with at least two years of follow-up. Patients were divided into groups based on the type of CNI treatment, including CsA (*n* = 60) and Tac (*n* = 68) groups. The study was conducted in accordance with local Good Clinical Practice guidelines and current legislations, while permission was obtained from the institutional ethics committee to use patient data for publication purposes (Date of Approval: 12/08/2014; Reference number/Protocol No: 2014–15/20).

### Assessments

Data on patient demographics (age, gender) and clinical characteristics (primary diagnosis, transplant type, diabetes mellitus treatment, post-transplant diabetes mellitus and hypertension) were retrieved from hospital records. Data on blood glucose (mg/dL) and creatinine (mg/dL) levels, estimated glomerular filtration rate (eGFR; mL/min/1.73 m2), total-cholesterol (mg/dL), low-density lipoprotein (LDL)-cholesterol (mg/dL), high-density lipoprotein (HDL)-cholesterol (mg/dL) and triglyceride (mg/dL) levels, systolic (SBP) and diastolic (DBP) blood pressure (mmHg) levels and anthropometric measurements (height [m], body weight [kg], BMI [kg/m2], body fat percentage [BF%, %], WaC [cm], HC [cm], WHR, wrist circumference [WrC, cm] and neck circumference [NC, cm]) were recorded in each patient both in the pre-transplant and post-transplant 4-year follow-up periods (1st, 3rd, 6th, 12th, 24th, 36th and 48th months). Post-transplant diabetes diagnosis was made using the American Diabetes Association diagnostic criteria; symptoms of diabetes (polyuria, polydipsia and unexplained weight loss) plus random plasma glucose ≥ 200 mg/dL or fasting plasma glucose ≥ 126 mg/dL or 2-h plasma glucose ≥ 200 mg/dL during an oral glucose tolerance test or HbA1c ≥ 6.5% [16]. Study parameters were compared in CsA and Tac treatment groups.

### Immunosuppressive therapy

All patients underwent the first kidney transplantation. For induction therapy, the patients received 20 mg of intravenous basiliximab (on days 0 and 4) or antithymocyte globulin-Fresenius (ATG, *n* = 15). The maintenance treatments consisted of CNI (CsA 5 mg/kg/d or Tac 0.15 mg/kg/d) and mycophenolic acid [mycophenolate mofetil (MMF) 2,000 mg/d or enteric-coated mycophenolate sodium (EC-MPS) 1,440 mg/d] with corticosteroids. 500 mg of methylprednisolone was given intravenously perioperatively and continued at 250 mg, 160 mg, 120 mg, and 80 mg consecutively for the next four days. Subsequently, patients received oral prednisolone (60 mg, 50 mg, 40 mg, and 30 mg daily). The daily oral prednisolone dose was reduced to 20 mg 1 month later, 10 mg 2 months later, and 5 mg 6 months later. We had either increased or decreased steroid doses in the following situations: low basal body weight (< 50 kg), development of de novo diabetes mellitus, increased insulin requirement in diabetics, high immunological risk, acute rejection, and premedication before ATG induction and plasmapheresis. We adjusted the dosages of CNIs to reach target trough levels; 200–300 ng/mL in the first three months and then 100–200 ng/mL for CsA, 8–12 ng/mL for the first three months, and then 5–8 ng/mL for Tac. We measured Tac drug levels by microparticle enzyme immunoassay (MEIA) method (Abbott IMx) and CsA drug levels by fluorescence polarization immunoassay (FPIA) method (Abbott TDx).

### Anthropometrics

The same researcher on all patients performed anthropometric measurements. BMI was calculated as weight in kilograms divided by the square of height in meters (kg/m^2^). Body composition was evaluated using the multi-frequency bioimpedance analysis method with a body composition monitor Tanita TBF-622 (Tanita Corporation, Tokyo). eGFR was calculated using the 2009 Chronic Kidney Disease Epidemiology Collaboration (CKD-EPI) creatinine equation [17]. 

### Statistical analysis

Data analysis was performed using IBM SPSS Statistics version 25.0 software (IBM Corporation, Armonk, NY, US). Kolmogorov–Smirnov and Levene tests were used to evaluate whether the normal distribution and homogeneity of variance assumptions were met. Categorical data were expressed as numbers (n) and percentages (%), while quantitative data were given as mean ± SD or median (25th—75th) percentiles. While the mean differences between groups were compared with Student’s t-test, the Mann–Whitney U test was applied to compare the continuous variables in which the parametrical test assumptions were not met. The intragroup comparisons from baseline to 48th month were performed using a paired t-test for normally distributed data and Wilcoxon signed ranks sum for non-normally distributed data. When appropriate, categorical variables were analysed with Pearson's χ2 or Fisher's exact test. Percentage change values from baseline to each follow-up period (at 1st, 3rd, …, 48th months) for each numerical variable in the Tac and CsA groups were calculated with the following formula, and the percentage change rates of groups were compared.

Percentage change = ([1st/3rd/…/48th month value – baseline value]/baseline value) × 100. The correlation between the percentage change values of the group variables was evaluated using Spearman’s correlation. Multiple logistic regression analyses via the Backwards LR procedure investigated the best independent predictor(s), which mainly affected the development of obesity in the 48^th^ month. Any variable whose univariable test had a p-value less than 0.25 was accepted as a candidate for the multivariable model. Odd’s ratios (OR), 95% confidence intervals (CI) and Wald statistics for each independent variable were calculated. For every single final model in multivariate analyses, the statistics of the Hosmer and Lemeshow goodness of fit test, Cox and Snell R^2^ and Nagelkerke R^2^ were also obtained. A *p*-value less than 0.05 was considered statistically significant.

## Results

### Demographic and clinical characteristics

**Fig. 1:**
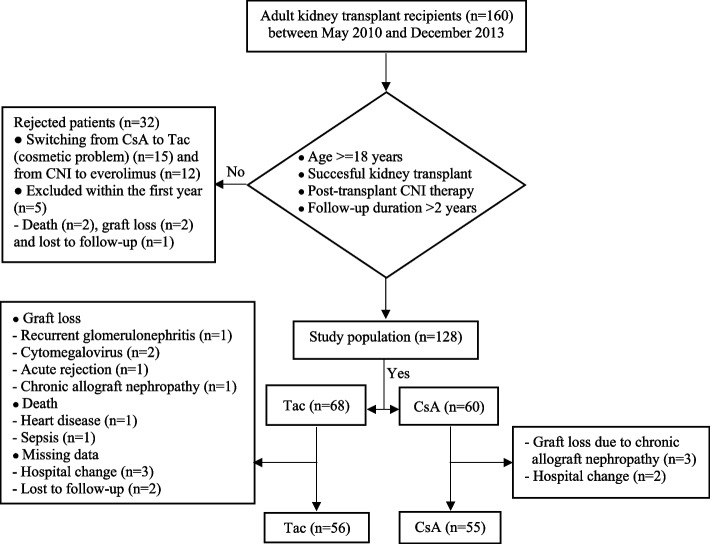
Flow chart of the study

Twelve patients in the Tac group and five in the CsA group could not complete the 48-month study period for different reasons (Fig. 1). The transplant age of the patients in the CsA group was higher compared to the patients in the Tac group (median 44.0 vs. 36.0 years, *p* = 0.02). No significant difference was noted between CsA and Tac groups regarding gender, dialysis features, transplant type, acute rejection, and cumulative prednisolone doses (Table 1). Pre-transplant blood pressures, anthropometric measurements and lipid values of both groups were comparable (*p* > 0.05). Only mean serum glucose levels in the CsA group were significantly higher than in the Tac group (107.9 ± 48.3 mg/dL vs. 89.6 ± 22.5 mg/dL, *p* = 0.012).

**Table 1:** Demographic, dialysis and transplant characteristics of patients in treatment groups

**Variables**	**CsA Group** **(*n* = 60)**	**Tac Group** **(*n* = 68)**	***p* value**
Transplant age (years)	44 (20–64)	36 (19–62)	0.020
Gender (female/male)	32/28	37/31	0.903
Primary etiology n (%)			
Diabetes mellitus	11 (18.3)	1 (1.5)	< 0.001
Hypertension	25 (41.7)	32 (47.1)	> 0.05
Glomerulonephritis	2 (3.3)	11 (16.2)	> 0.05
Polycystic kidney diseases	5 (8.3)	3 (4.4)	> 0.05
Nephrolithiasis	4 (6.7)	1 (1.5)	> 0.05
Obstructive nephropathy	2 (3.3)	6 (8.8)	> 0.05
Amyloidosis	1 (1.7)	2 (2.9)	> 0.05
Unknown etiology	10 (16.7)	12 (17.6)	> 0.05
Dialysis modality			
Pre-emptive	8 (13.3)	14 (20.6)	0.759
Hemodialysis	38 (63.3)	38 (55.9)	
Peritoneal dialysis	8 (13.3)	9 (13.2)	
Both dialysis	6 (10.0)	7 (10.3)	
Dialysis duration (month)	66 (0:220)	53 (0:228)	0.851
Transplant type (living/deceased)	27/33	34/34	0.319
Donor age (years)	49 (22–80)	50 (17–85)	0.822
Cold ischemia time (hour)	9.5 (1:20)	4.5 (1:16)	0.439
HLA mismatch	3.13 ± 1.19	3.42 ± 1.05	0.144
Induction treatment (ATG/basiliximab)	2/58	13/55	0.006
Immunosuppressive regimen n (%)			0.889
CsA + EC-MPS + prednisolone	21 (35)	-	
CsA + MMF + prednisolone	39 (65)	-	
Tac + EC-MPS + prednisolone	-	23 (33.8)	
Tac + MMF + prednisolone	-	45 (66.2)	
Pre-operative steroid use n (%)	5 (8.3)	11 (16.2)	0.284
Cumulative dose of prednisolone (mg)	11,187 ± 1,529	10,884 ± 1,526	0.289
Cytomegalovirus history n (%)	17 (28.3)	18 (26.5)	0.845
BK virus n (%)	8 (13.3)	6 (8.8)	0.572
Acute rejection n (%)	4 (6.7)	9 (13.2)	0.220
Delayed graft function n (%)	19 (31.7)	17 (25)	0.403

Data were expressed as mean ± SD (standard deviation) or n (%). CsA: cyclosporine A, Tac: tacrolimus, ATG: antithymocyte globulin, EC-MPS: enteric-coated mycophenolate sodium, MMF: mycophenolate mofetil

### Anthropometrics

**Fig. 2:**
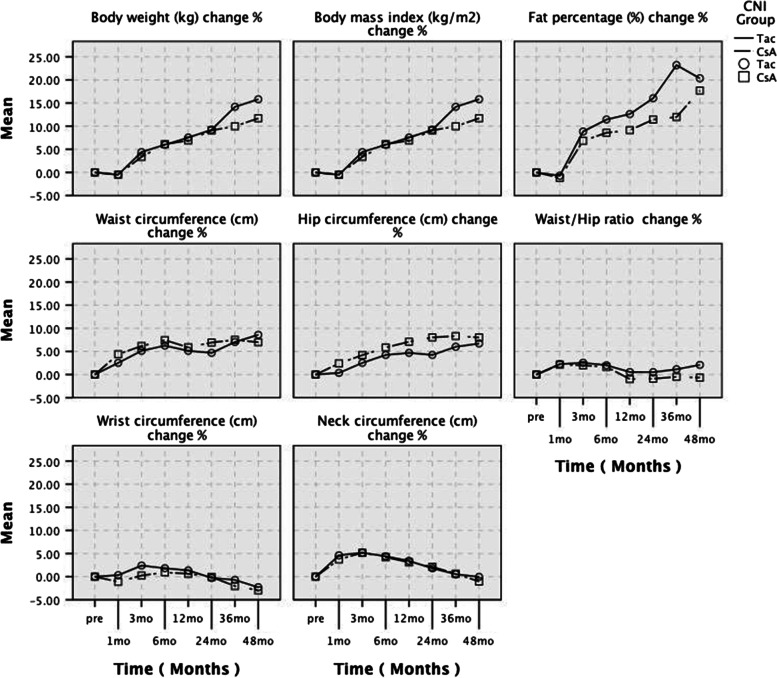
Comparison of percentage changes in anthropometric measurements between both groups

Mean weight gains were 4.26 ± 6.57 and 4.37 ± 6.52 kg in the 12^th^ month, 5.72 ± 7.10 and 5.51 ± 7.05 kg in the 24^th^ month, 6.32 ± 7.71 and 8.45 ± 8.81 kg in 36^th^ month and 7.39 ± 8.16 and 9.63 ± 8.24 kg in the 48^th^ month in the CsA and Tac groups, respectively (*p* > 0.05). Compared with the pre-transplant values, mean body weight and BMI values increased in the 3^rd^, 6^th^, 12^th^, 24^th^, 36^th^ and 48^th^ months post-transplant, and WaC and HC values in the 1^st^, 3^rd^, 6^th^, 12^th^, 24^th^, 36^th^ and 48^th^ months post-transplant (*p* values ranged from < 0.05 to < 0.001). Post-transplant WHR values increased in the 1^st^ and 3^rd^ months in the CsA group and in the 1^st^, 3^rd^ and 6^th^ months in the Tac group, and post-transplant NC values in the 1^st^, 3^rd^, 6^th^ and 12^th^ months in the CsA group and in the 1^st^, 3^rd^, 6^th^, 12^th^ and 24^th^ months in the Tac group (*p* values ranged from < 0.05 to < 0.001). Compared with the pre-transplant values, a significant increase in post-transplant BF% values was noted between the 3^rd^ and 24^th^ months in the CsA group and between the 24^th^ and 48^th^ months in the Tac group (*p* values ranged from < 0.05 to < 0.001). Compared with the pre-transplant values, WrC values increased significantly in the post-transplant 3^rd^ and 6^th^ months in the Tac group. In contrast, significant decreases were noted in WrC values in the post-transplant 48^th^ month in both CsA (*p* < 0.001) and Tac (*p* < 0.05) groups (Table 2). HC percentage changes from the pre-transplant period to 1^st^ (2.40 ± 6.16% vs. 0.36 ± 4.90%, *p* = 0.039), 12^th^ (7.08 ± 8.27% vs. 4.68 ± 6.81%, *p* = 0.048) and 24^th^ (8.03 ± 8.75% vs. 4.23 ± 8.05%, *p *= 0.012) months were significantly higher in CsA than in the Tac group. However, no significant difference was observed between the CsA and Tac groups at each time point regarding the percentage change values ​​for other anthropometric parameters (Fig. 2).

**Table 2:** Comparison of effects of cyclosporine A and tacrolimus on anthropometric measurements

		**Pre-transplant**	**1**^**st**^** month**	**3**^**rd**^** month**	**6**^**th**^** month**	**12**^**th**^** month**	**24**^**th**^** month**	**36**^**th**^** month**	**48**^**th**^** month**
**Variables**	CsA (n)	60	60	60	60	60	60	56	55
	Tac (n)	68	68	68	68	68	68	60	56
Body weight (kg)	CsA	63.5 ± 11.9	63.1 ± 12.3	65.4 ± 12.0*	67.2 ± 12.9**	67.8 ± 13.4**	69.2 ± 14.4**	69.9 ± 15.4**	70.9 ± 15.7**
	Tac	62.7 ± 15.3	62.1 ± 14.3	64.9 ± 14.1*	66.0 ± 15.0**	67.1 ± 16.1**	68.2 ± 17.1**	71.1 ± 19.1**	72.8 ± 18.9**
Body mass index (kg/m^2^)	CsA	24.0 ± 4.4	23.8 ± 4.4	24.7 ± 4.2*	25.3 ± 4.4**	25.5 ± 4.6**	26.1 ± 5.1**	26.4 ± 5.4**	26.8 ± 5.6**
	Tac	23.7 ± 4.8	23.4 ± 4.3	24.5 ± 4.3*	24.9 ± 4.5**	25.3 ± 4.8**	25.7 ± 5.1**	27.0 ± 5.8**	27.7 ± 5.9**
Body fat percentage (%)	CsA	23.4 ± 7.8	23.1 ± 8.3	24.8 ± 8.7*	25.0 ± 8.7*	25.1 ± 9.1*	25.3 ± 8.9*	25.7 ± 10.0*	27.0 ± 9.6**
	Tac	23.7 ± 9.9	23.3 ± 10.1	24.6 ± 9.3	24.9 ± 9.3	25.1 ± 9.8	26.0 ± 10.4*	27.6 ± 11.1*	27.3 ± 10.8*
Waist circumference (cm)	CsA	85.5 ± 10.9	89.1 ± 11.4*	90.6 ± 11.5**	91.7 ± 12.3**	90.4 ± 13.3**	91.3 ± 14.1**	91.9 ± 15.7**	91.6 ± 17.3**
	Tac	84.7 ± 12.9	86.4 ± 11.8*	88.5 ± 12.1**	89.5 ± 12.2**	88.7 ± 13.5**	88.5 ± 15.5*	90.3 ± 16.8**	92.3 ± 16.1**
Hip circumference (cm)	CsA	93.8 ± 10.0	95.7 ± 9.1*	97.4 ± 9.1*	98.9 ± 9.3**	100.0 ± 9.8**	100.8 ± 9.7**	100.8 ± 10.0**	100.6 ± 9.8**
	Tac	94.8 ± 9.6	94.9 ± 9.1*	97.0 ± 9.1*	98.5 ± 9.4**	99.0 ± 10.1**	98.5 ± 9.9**	99.7 ± 9.9**	100.9 ± 11.0**
Waist-to-hip ratio	CsA	0.91 ± 0.06	0.93 ± 0.07*	0.92 ± 0.06*	0.92 ± 0.07	0.90 ± 0.07	0.90 ± 0.08	0.90 ± 0.10	0.90 ± 0.11
	Tac	0.89 ± 0.08	0.90 ± 0.07*	0.91 ± 0.06*	0.90 ± 0.06*	0.89 ± 0.07	0.89 ± 0.09	0.90 ± 0.11	0.91 ± 0.10
Wrist circumference (cm)	CsA	16.7 ± 1.3	16.5 ± 1.4	16.7 ± 1.3	16.8 ± 1.5	16.8 ± 1.7	16.7 ± 1.5	16.3 ± 1.5	16.1 ± 1.2**
	Tac	16.3 ± 1.7	16.4 ± 1.7	16.7 ± 1.6*	16.6 ± 1.5*	16.5 ± 1.6	16.3 ± 1.6	16.1 ± 1.6	15.9 ± 1.6*
Neck circumference (cm)	CsA	35.7 ± 2.7	37.0 ± 2.8**	37.5 ± 2.9**	37.1 ± 3.2**	36.7 ± 3.4*	36.4 ± 3.3*	35.9 ± 3.4	35.2 ± 3.4
	Tac	35.3 ± 3.6	36.9 ± 3.3**	37.1 ± 3.3**	36.8 ± 3.3**	36.5 ± 3.6**	35.9 ± 4.0	35.6 ± 4.0	35.6 ± 4.6

Data were expressed as mean ± SD. *CsA* cyclosporine-A, *Tac* tacrolimus. ^*^p < 0.05 and ^**^p < 0.001; compared to intragroup pre-transplant values

### Blood pressure and graft function

**Fig. 3:**
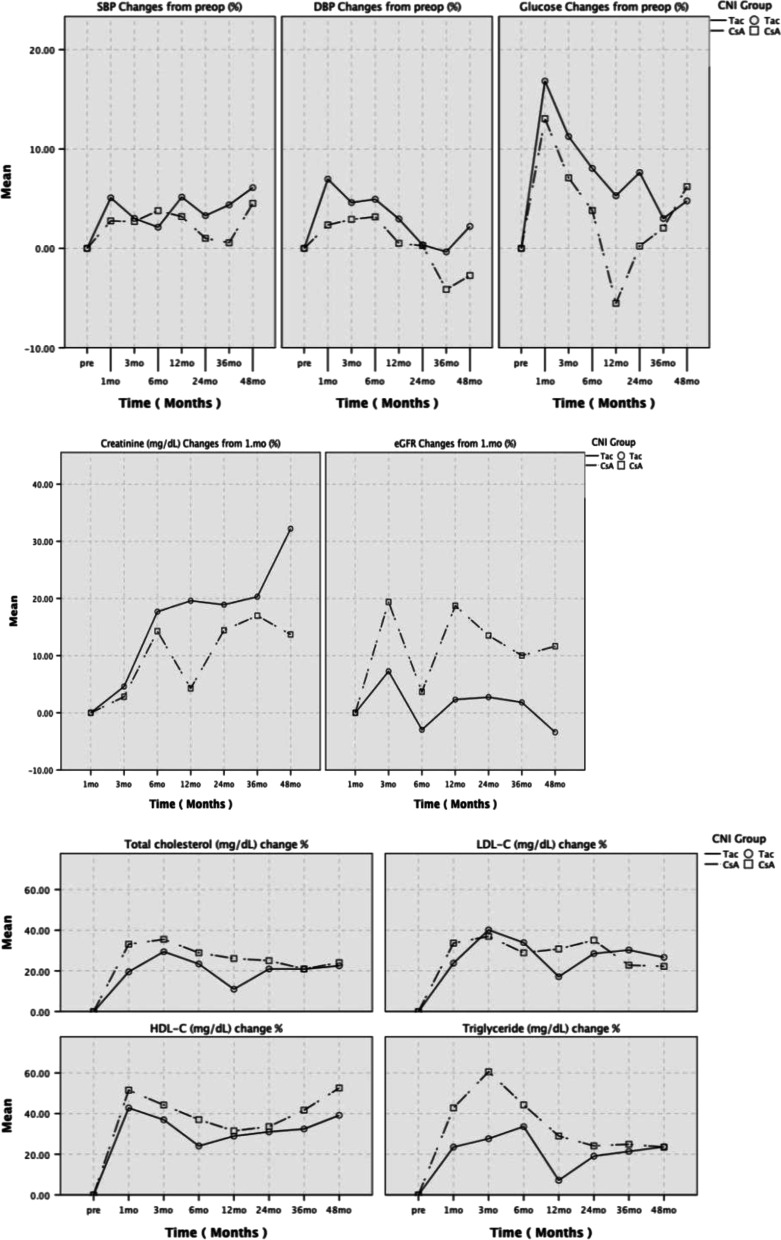
Comparison of percentage changes in blood pressure, serum glucose, serum creatinine, eGFR and lipid profile between both groups

Compared to pre-transplant values, DBP decreased significantly in the 36^th^ month in the CsA group (*p* = 0.022) and increased in the 1^st^ month in the Tac group (*p* = 0.019). In the Tac group, eGFR values at the 6^th^ (*p* < 0.001) and 48^th^ months (*p* = 0.008) were significantly lower than the 1^st^-month values. In the CsA and Tac groups, there was no significant change in SBP and serum creatinine values at each time point compared with pre-transplant and post-transplant 1-month values, respectively (Table 3). In both groups, no significant difference was observed between percentage changes in BPs, serum creatinine and eGFR values (Fig. 3).

**Table 3:** Comparison of effects of cyclosporine A and tacrolimus on blood pressure and graft function tests

		**Pre-transplant**	**1**^**st**^** month**	**3**^**rd**^** month**	**6**^**th**^** month**	**12**^**th**^** month**	**24**^**th**^** month**	**36**^**th**^** month**	**48**^**th**^** month**
**Variables**	CsA (n)	60	60	60	60	60	60	56	55
	Tac (n)	68	68	68	68	68	68	60	56
Systolic BP (mmHg)	CsA	127.4 ± 23.4	127.7 ± 13.6	127.0 ± 13.0	128.5 ± 15.6	127.7 ± 15.1	124.6 ± 13.8	125.0 ± 14.0	128.8 ± 18.8
	Tac	122.2 ± 16.6	126.6 ± 14.5	123.9 ± 13.9	123.2 ± 15.2	126.4 ± 16.7	124.1 ± 18.3	125.9 ± 20.7	127.2 ± 17.4
Diastolic BP (mmHg)	CsA	79.1 ± 11.6	79.5 ± 8.3	79.8 ± 8.7	79.8 ± 8.9	78.0 ± 7.9	77.6 ± 9.4	75.1 ± 11.3*	75.8 ± 13.5
	Tac	76.1 ± 10.0	80.1 ± 9.6*	78.5 ± 8.6	78.8 ± 9.2	77.3 ± 9.5	75.1 ± 10.9	75.3 ± 13.5	76.2 ± 12.2
Hemoglobin (g/dL)	CsA	11.59 ± 1.88	12.0 ± 1.80	12.82 ± 2.44**	12.73 ± 2.15**	12.97 ± 1.95**	12.77 ± 1.76**	12.82 ± 1.76**	12.54 ± 1.79*
	Tac	11.16 ± 1.89	11.5 ± 1.51	12.91 ± 1.88**	12.67 ± 1.97**	12.96 ± 1.96**	13.11 ± 1.57**	13.22 ± 1.61**	13.2 ± 1.51**
Albumin (g/dL)	CsA	3.47 ± 0.63	3.44 ± 0.57	3.80 ± 0.48**	3.92 ± 0.43**	4.06 ± 0.31**	4.11 ± 0.39**	4.10 ± 0.34**	4.14 ± 0.31**
	Tac	3.52 ± 0.64	3.76 ± 0.44*	4.07 ± 0.58**	4.06 ± 0.55**	4.15 ± 0.34**	4.20 ± 0.39**	4.21 ± 0.35**	4.22 ± 0.38**
Creatinine (mg/dL)	CsA	8.83 ± 3.2	1.33 ± 0.5	1.27 ± 0.4	1.41 ± 0.5	1.27 ± 0.4	1.37 ± 0.7	1.44 ± 0.9	1.36 ± 0.5
	Tac	8.83 ± 2.5	1.32 ± 0.8	1.28 ± 0.5	1.42 ± 0.5	1.41 ± 0.6	1.39 ± 0.6	1.41 ± 0.6	1.52 ± 0.7
eGFR (mL/min/1.73 m^2^)	CsA	7.13 ± 3.7	63.8 ± 26.1	66.0 ± 24.3	58.9 ± 21.3	64.9 ± 19.7	62.5 ± 21.0	60.2 ± 23.5	60.8 ± 21.1
	Tac	7.29 ± 3.0	69.1 ± 27.6	68.5 ± 25.2	60.6 ± 24.0^q^	63.9 ± 26.2	63.6 ± 26.0	63.4 ± 26.8	59.0 ± 22.9^qq^

Data were expressed as mean ± SD. *CsA* cyclosporine A, *Tac* tacrolimus, *BP* blood pressure, *eGFR* estimated glomerular filtration rate. ^*^p < 0.05 and ^**^p < 0.001, compared to pre-transplant values; ^q^*p* < 0.001 and ^qq^*p* < 0.05, compared to post-transplant 1-month values

Compared with the pre-transplant values, mean haemoglobin values increased significantly in both groups from the 3^rd^ month. Compared to baseline, serum albumin levels increased significantly from the 1^st^ month in the Tac group and the 3^rd^ month in the CsA group (Table 3). The haemoglobin increase rate at 36^th^ (22.1 ± 23.9% vs. 11.7 ± 20.4%, *p* = 0.020) and 48^th^ (20.5 ± 22.6% vs. 9.22 ± 18.9%, *p* = 0.004) months in the Tac group were significantly higher than in the CsA group.

### Serum glucose and lipid measurements

Compared to pre-transplant values, blood glucose levels showed a significant decrease in the 12^th^ month in the CsA group (*p* = 0.008) and a significant increase in the 1^st^ month in the Tac group (*p* = 0.005) (Table 4). The mean serum glucose percentage changes in the Tac group were higher at the 6^th^ (8.04 ± 33.1% vs. 3.80 ± 52.4%, *p* = 0.049), 12^th^ (5.29 ± 26.0% vs. –5.53 ± 29.3%, *p* = 0.030) and 24^th^ (7.63 ± 31.0% vs. 0.23 ± 44.4%, *p* = 0.045) months than in the CsA group (Fig. 3).

**Table 4:** Comparison of effects of cyclosporine A and tacrolimus on metabolic parameters

		**Pre-transplant**	**1**^**st**^** month**	**3**^**rd**^** month**	**6**^**th**^** month**	**12**^**th**^** month**	**24**^**th**^** month**	**36**^**th**^** month**	**48**^**th**^** month**
**Variables**	CsA (n)	60	60	60	60	60	60	56	55
	Tac (n)	68	68	68	68	68	68	60	56
Glucose (mg/dL)	CsA	107.9 ± 48.3	108.1 ± 34.3	105.8 ± 33.6	100.2 ± 47.4	92.3 ± 23.2*	96.3 ± 32.8	97.5 ± 42.4	101.8 ± 43.2
	Tac	89.6 ± 22.5	100.4 ± 23.6*	95.8 ± 22.5	92.0 ± 19.4	90.4 ± 16.9	92.6 ± 24.9	89.2 ± 25.2	90.1 ± 18.0
Total-cholesterol (mg/dL)	CsA	183.9 ± 57.6	232.5 ± 74.5**	226.9 ± 52.1**	219.9 ± 51.3**	214.9 ± 49.3**	213.1 ± 51.7*	203.9 ± 50.4*	214.7 ± 55.6*
	Tac	173.0 ± 49.7	201.4 ± 55.0**	213.7 ± 46.8**	203.3 ± 56.6**	182.8 ± 38.2*	195.9 ± 44.9**	200.9 ± 54.3*	200.1 ± 50.6*
HDL-cholesterol (mg/dL)	CsA	40.1 ± 14.4	55.3 ± 18.5**	50.9 ± 18.0**	49.1 ± 13.8**	48.1 ± 12.4**	47.8 ± 13.6**	50.4 ± 12.6**	53.9 ± 14.2**
	Tac	36.8 ± 12.3	49.8 ± 17.5**	47.8 ± 16.6**	42.8 ± 14.2**	44.1 ± 12.9**	45.1 ± 12.8**	45.2 ± 12.7**	47.3 ± 13.6**
LDL-cholesterol (mg/dL)	CsA	113.5 ± 43.7	144.6 ± 63.2**	140.7 ± 47.4**	134.8 ± 43.8*	136.0 ± 44.2*	150.0 ± 120.0*	124.8 ± 42.4	129.3 ± 48.6
	Tac	102.7 ± 40.7	117.9 ± 43.8*	130.9 ± 38.8**	124.6 ± 45.1*	109.4 ± 31.1	118.9 ± 37.3**	122.8 ± 46.2*	117.6 ± 43.2*
Triglyceride (mg/dL)	CsA	150.9 ± 82.4	162.5 ± 90.5	176.2 ± 87.6	170.1 ± 82.8	159.1 ± 84.3	149.6 ± 94.9	143.1 ± 70.8	153.0 ± 80.6
	Tac	167.1 ± 105.1	168.0 ± 70.0	175.0 ± 85.0	179.1 ± 88.2	146.2 ± 75.4	154.5 ± 75.6	165.8 ± 90.3	175.7 ± 110.1

Data were expressed as mean ± SD. *CsA* cyclosporine A, *Tac* tacrolimus, *LDL* low-density lipoprotein, *HDL* high-density lipoprotein. ^*^p < 0.05 and ^**^p < 0.001; compared to intragroup pre-transplant values

Total- and HDL-cholesterol values increased significantly in both groups compared to the pre-transplant values during the 48-month follow-up period. LDL-cholesterol levels increased between 1^st^ and 24^th^ months in the CsA group and between 1^st^ and 48^th^ months in the Tac group after transplantation (*p* values ranged from < 0.05 to < 0.001). No significant change was observed in triglyceride levels in both groups (Table 4).

Compared with the pre-transplant values, percentage changes in the total cholesterol at 1^st^ (33.11 ± 42.01% vs. 19.56 ± 28.73%, *p *= 0.036) and 12^th^ (26.10 ± 44.35% vs. 10.99 ± 30.85%, *p* = 0.027) months were significantly higher in the CsA group than in the Tac group. At the same time, no significant difference was observed between the CsA and Tac groups in terms of percentage changes in HDL-cholesterol, LDL-cholesterol and triglyceride (Fig. 3).

Correlations of percentage changes between lipid values and anthropometric measurements were examined. In the Tac group, LDL-cholesterol was inversely correlated with BF% in the 1^st^ month. The positive correlations were as follows: total-cholesterol with WaC and HC in the 12^th^ month and WaC in the 36^th^ month; triglyceride with weight, BMI, WaC, HC and BF% in the 12^th^ month and BF% in the 36^th^ month; LDL-cholesterol with WaC and NC in the 36^th^ month. In the CsA group, HDL-cholesterol was inversely correlated with WaC in the 24^th^ month, WHR in the 36^th^ month and WrC in the 48^th^ month. The positive correlations were as follows: total-cholesterol with WrC in the 6^th^ month; HDL-cholesterol with weight and BMI in the 1^st^ month, and weight, BMI and WrC in the 3^rd^ month; triglyceride with weight, BMI and BF% in the 6^th^ month, WHR, WrC and NC in the 24^th^ month, NC in the 36^th^ and 48^th^ months; LDL-cholesterol WrC and NC in the 6^th^ months (Table 5).

**Table 5:** Significant correlations in percentage changes between anthropometric measurements (body fat percentage, weight, BMI, waist, hip, wrist and neck circumferences, waist-hip ratio) with lipid values in cyclosporine A and tacrolimus groups throughout the study

Lipid parameters	CsA group	Tac group
Total-cholesterol	Mo 6: WrC (r: 0.272, *p* = 0.037)	Mo 12: WaC (r: 0.267, *p* = 0.030), HC (r: 0.339, *p* = 0.005)
		Mo 36: WaC (r: 0.339, *p* = 0.011)
HDL-cholesterol	Mo 1: W (r: 0.330, *p* = 0.011), BMI (r: 0.331, *p* = 0.011)	-
	Mo 3: W (r: 0.274, *p* = 0.040), BMI (r: 0.274, *p* = 0.040), WrC (r: 0.277, *p* = 0.037)	
	Mo 24: WaC (r: -0.272, *p* = 0.035)	
	Mo 36: WHR (r: -0.287, *p* = 0.032)	
	Mo 48: WrC (r: -0.291, *p* = 0.035)	
Triglyceride	Mo 6: W (r: 0.319, *p* = 0.014), BMI (r: 0.319, *p* = 0.014), BF% (r: 0.411, *p* = 0.001)	Mo 12: W (r: 0.369, *p* = 0.002), BMI (r: 0.369, *p* = 0.002), WaC (r: 0.246, *p* = 0.047), HC (r: 0.316, *p* = 0.010), BF% (r: 0.265, *p* = 0.032)
	Mo 24: WHR (r: 0.270, *p* = 0.037), WrC (r: 0.327, *p* = 0.011), NC (r: 0.308, *p* = 0.017)	Mo 36: BF% (r: 0.269, *p* = 0.047)
	Mo 36: NC (r: 0.326, *p* = 0.014)	
	Mo 48: NC (r: 0.277, *p* = 0.043)	
LDL-cholesterol	Mo 6: WrC (r: 0.262, *p* = 0.045), NC (r: 0.308, *p* = 0.018)	Mo 1: BF% (r: -0.261, *p* = 0.034)
		Mo 36: WaC (r: 0.276, *p* = 0.039), NC (r: 0.398, *p* = 0.002)

*CsA* cyclosporine A, *Tac* tacrolimus, *W* weight, *BMI* body mass index, *WaC* waist circumference, *HC* hip circumference, *WHR* waist-to-hip ratio, *WrC* wrist circumference, *NC* neck circumference

### Clinical events and comorbidities

While the rates of patients with dyslipidemia before transplantation were similar in both groups, the rate of patients with dyslipidemia after transplantation was higher in the CsA group (38.3% vs. 20.6%, *p* = 0.027). There was no significant difference in the rates of hypertension, coronary artery disease and cerebrovascular accident between CsA and Tac groups in the pre-transplant period. Post-transplant comorbidity rates were also comparable between the CsA and Tac groups (Table 6).

**Table 6:** Frequency of comorbidities and clinical events before and after transplantation in treatment groups

		**CsA Group** **(*n* = 60)**	**Tac Group** **(*n* = 68)**	***p* value**
Clinical events n (%)				
Hypertension	Pre-op	45 (75.0)	42 (61.8)	0.109
	Post-op	3 (5.0)	8 (11.8)	0.216
Dyslipidemia	Pre-op	7 (11.7)	10 (14.7)	0.795
	Post-op	23 (38.3)	14 (20.6)	0.027
Diabetes mellitus	Pre-op	11 (18.3)	1 (1.5)	0.001
	Post-op	5 (8.3)	13 (19.1)	0.125
Cerebrovascular event	Pre-op	2 (3.3)	0 (0)	0.218
	Post-op	1 (1.7)	0 (0)	0.469
Coronary artery disease	Pre-op	3 (5.0)	3 (4.4)	1.000
	Post-op	4 (6.7)	5 (7.4)	1.000
Heart failure	Pre-op	1 (1.7)	1 (1.5)	1.000
	Post-op	1 (1.7)	0 (0)	0.469
Urinary tract infection	Post-op	18 (30.0)	24 (35.2)	0.524
History of comorbidity n (%)				
Smoking		13 (21.7)	15 (22.1)	0.957
Alcohol use		1 (1.7)	2 (2.9)	1.000
Regular exercise habit		7 (11.7)	9 (13.2)	1.000
Diabetes mellitus		16 (26.7)	14 (20.6)	0.418
Insulin use		15 (25.0)	8 (11.8)	0.066
Dyslipidemia		30 (50.0)	25 (36.8)	0.131
Hypertension		48 (80.0)	50 (73.5)	0.388
Coronary artery disease		7 (11.7)	7 (10.3)	1.000
Myocardial infarction		7 (4.7)	6 (8.8)	0.771
Coronary angiography		11 (18.3)	9 (13.2)	0.472
Coronary angioplasty/stent		6 (10.0)	4 (5.9)	0.514
Coronary bypass surgery		2 (3.3)	3 (4.4)	1.000
Heart failure		2 (3.3)	1 (1.5)	0.600
Heart valve disease		1 (1.7)	2 (2.9)	1.000
Arrhythmia		2 (3.3)	3 (4.4)	1.000
Peripheral artery disease		2 (3.3)	1 (1.5)	0.600
Chronic lung disease		5 (8.3)	4 (5.9)	0.733
Cerebrovascular event		3 (5.0)	0 (0)	0.100
Hemorrhage		1 (1.7)	-	
Infarct		2 (3.3)	-	
Malignancy		1 (1.7)	0 (0)	0.469
Hepatitis B virus n (%)		3 (5.0)	5 (7.4)	0.722
Hepatitis C virus n (%)		4 (6.7)	2 (2.8)	0.418

Data were expressed as n (%). *CsA* cyclosporine A, *Tac* tacrolimus

The prevalence of diabetes mellitus was higher in the CsA group than in the Tac group (18.3% vs. 1.5%, *p* < 0.001). In the pre-transplant period, there were eleven patients with diabetes mellitus (6 type 1, 5 type 2) in the CsA group and one patient with diabetes mellitus (type 1) in the Tac group. Insulin therapy continued during the post-operative period in 7 patients with type 1 diabetes and two patients with type 2 diabetes. Insulin therapy was started in the post-transplant period in 2 of the other type 2 diabetes patients who used oral antihyperglycemic drugs and in one patient who only took a diet before transplantation.

De novo diabetes mellitus developed in 5 patients (8.3%) in the CsA group and 13 patients (19.1%) in the Tac group (*p* = 0.08). Four patients in the CsA group continued on low-dose insulin therapy (10–14 U insulin glargine and/or 18–30 U/day insulin aspart) throughout the study. We gave insulin to another patient for only one year and stopped the treatment. Seven patients in the Tac group received post-transplant insulin therapy. While insulin therapy was continued in three of them, the need for insulin disappeared in four within the first six months. Six patients used oral antihyperglycemic drug therapy, and three were subsequently followed up with diet alone.

Of the 30 diabetic patients, 23 used post-transplant insulin therapy, and five did not require treatment other than diet after the first year. We performed two subgroup analyses. The first analysis compared anthropometric measurements in diabetic (*n* = 30) and non-diabetic (*n* = 98) patients, and the second analysis in insulin-using (*n* = 23) and non-insulin-using (*n* = 105) patients. In the first analysis, only baseline BF% values were significantly higher in patients with diabetes than in non-diabetics (26.65 ± 7.84% vs. 22.66 ± 9.12%, *p* = 0.023). The rates of increase in NC (6.32 ± 7.98% vs. 2.32 ± 6.71%, *p* = 0.009) in the 12^th^ month in diabetic patients and BF% at the 24^th^ (2.58 ± 32.94% vs. 17.33 ± 35.0%, *p* = 0.030) and 36^th^ months (5.75 ± 34.99% vs. 20.89 ± 37.8%, *p* = 0.031) in non-diabetics were significantly higher than the other group. In the second analysis, although the baseline anthropometric measurements were comparable, the increase rates in NC from the 3^rd^ month to the 24^th^ month (38.13 ± 2.76% vs. 37.13 ± 3.22%, *p* = 0.023; 37.91 ± 3.13% vs. 36.81 ± 3.32%, *p* = 0.015; 38.0 ± 3.71% vs. 36.34 ± 3.48%, *p* = 0.001 and 37.13 ± 4.18% vs. 36.01 ± 3.62%, *p* = 0.027, respectively) and WaC in the 12^th^ month (9.71 ± 10.16% vs. 4.55 ± 9.39%, *p* = 0.020) were significantly higher in patients who used insulin than in those who did not.

The frequency of urinary tract infection, which is common after transplantation and complicates glycemic control in diabetic patients, was evaluated. In the first year after transplantation, urinary tract infection developed in 35.2% of the patients in the Tac group (*n* = 24), while in 30% of the CsA group (*n* = 18) (*p* = 0.524).

### Risk factors analysis

The patients were divided into obese and non-obese groups according to the cut-off values for BMI (≥ 30 kg/m^2^), BF% (> 25% in males, > 35% in females), WaC (> 102 cm in males, > 88 cm in females) and WHR (> 0.9 in males, > 0.8 in females) [18, 19]. In the above four categories, after 48 months of follow-up, the factor(s) that were the most statistically determinant of the development of obesity was investigated with multivariate retrospective stepwise elimination (Backward LR) logistic regression analyses. As a result of univariate statistical analyses, all variables found to be *p* < 0.25 were included in the regression models as candidate risk factors. Only one of the variable pairs in which the multicollinearity problem was seen was included in the model, and the final model results for each primary outcome (BMI, BF%, WaC, WHR) as a result of the retrospective elimination procedure were given in Table 7.

**Table 7:** Multivariate logistic regression analysis of independent risk factors affecting the development of obesity at 48^th^ months (*n* = 109)

Reference category	OR	95% CI for OR	Wald	*P*-value
**Body mass index**				
Age	0.904	0.836–0.978	6.331	0.012
Baseline body mass index	2.099	1.535–2.870	21.578	< 0.001
Not to exercise	30.939	1.218–785.832	4.324	0.038
Cox and Snell *R*^2^ = 0.459, Nagelkerke *R*^2^ = 0.658, χ2 for Hosmer and Lemeshow goodness of fit test: 8.056 and *p* = 0.428				
**Body fat percentage**				
Baseline body mass index	1.887	1.451–2.455	22.383	< 0.001
Baseline wrist circumference	0.304	0.149–1.986	10.565	< 0.001
Baseline neck circumference	1.499	1.132–1.986	7.966	0.005
GFR level at the 1^st^ month	1.048	1.017–1.079	9.367	0.002
Not to exercise	30.726	2.247–420.115	6.588	0.010
Cox and Snell *R*^2^ = 0.491, Nagelkerke *R*^2^ = 0.665, χ2 for Hosmer and Lemeshow goodness of fit test: 10.551 and *p* = 0.228				
**Waist circumference**				
Baseline body mass index	1.493	1.110–2.007	7.036	0.008
Baseline waist circumference	1.185	1.063–1.321	9.445	0.002
Baseline wrist circumference	0.506	0.287–0.890	5.586	0.018
Cox and Snell *R*^2^ = 0.519, Nagelkerke *R*^2^ = 0.697, χ2 for Hosmer and Lemeshow goodness of fit test: 6.111 and p = 0.635				
**Waist-to-hip ratio**				
Baseline waist circumference	1.117	1.053–1.186	13.246	< 0.001
Not to exercise	4.751	1.080–20.894	4.252	0.039
History of dyslipidemia	4.606	1.357–15.631	6.001	0.014
Cox and Snell *R*^2^ = 0.316, Nagelkerke *R*^2^ = 0.457, χ2 for Hosmer and Lemeshow goodness of fit test: 9.600 and, *p* = 0.294				

*OR* Odd’s ratio, *CI* confidence interval

After 48 months of follow-up, the factors that were the most statistically determinant of the development of obesity in terms of BMI were having a high basal BMI, being young, and not exercising. The higher the basal BMI, the higher the probability of obesity independent of other factors. When adjustment was made between age and the development of obesity according to other factors, there was a statistically significant inverse association. Finally, it was determined that not exercising significantly increased the development of obesity.

After 48 months of follow-up, the most statistically determinant factors on the development of obesity in BF% were baseline BMI, baseline WrC, GFR level measured at one month, baseline NC, and not exercising. The higher the baseline BMI, the higher the probability of obesity independent of other factors. When adjustment for other factors was made between baseline WrC and the development of obesity, there was a statistically significant inverse association. As the GFR level increased in the first month, the probability of obesity independent of other factors also increased significantly. When adjusted for other factors, the increase in baseline NC significantly increased the development of obesity. Finally, it was determined that not exercising significantly increased the development of obesity.

After 48 months of follow-up, the most statistically determinant factors on the development of obesity in terms of WaC were, respectively, basal WaC, basal BMI and basal WrC. The higher the baseline WaC, the higher the probability of obesity independent of other factors. When adjustment was made for other factors, the increase in basal BMI increased the development of obesity statistically. When adjustment was made between the baseline WrC and the development of obesity according to other factors, there was a statistically significant inverse association.

After 48 months of follow-up, the most statistically determinant factors on the development of obesity in terms of WHR were baseline WaC, history of dyslipidemia and not exercising. The wider the baseline WaC, the higher the probability of obesity independent of other factors. When adjustment was made for other factors, the probability of developing obesity was statistically significantly higher in those with a history of dyslipidemia compared to those without a history of dyslipidemia. Finally, it was determined that not exercising significantly increased the development of obesity.

## Discussion

Cardiovascular diseases are still the main cause of death after kidney transplantation. In addition to the classical risk factors (obesity, diabetes mellitus, hypertension, hyperlipidemia, smoking, etc.), dialysis periods before transplantation, graft function after transplantation, proteinuria, acute rejection episodes, post-transplant diabetes, hyperhomocysteinemia and immunosuppressive drugs are also important risk factors [20].

An increase in body weight is commonly observed after transplantation [21]. The main factors underlying the post-transplant weight gain are the recovery of uremic elements, decreased diet restrictions, and increased appetite after the transplantation and immunosuppressive treatments [22]. In our cohort, weight gains were comparable in the CsA and Tac groups (median 3.75 and 4.15 kg in the 12^th^ month, 5.60 and 4.95 kg in the 24^th^ month, 6.25 and 8.95 kg in 36^th^ month and 7.0 and 10.25 kg in the 48^th^ month, respectively). Localization of adipose tissue plays a role in the development of many diseases [23]. High BMI (≥ 30 kg/m^2^) and BF% (> 25% in men, > 35% in women) indicate general obesity [18]. However, both do not provide information about where adiposity is localized in the body. BMI does not help differentiate muscle and fat tissue, nor does it supply adequate information about peripheral and central adiposity. Therefore, WaC (> 102 cm in men, > 88 cm in women), HC and WHR (> 0.9 in men, > 0.8 in women) have become more commonly used anthropometric indices in recent years [19]. Abdominal obesity is associated with insulin resistance and metabolic syndrome and determines cardiovascular risk. WaC and WHR are widely used as abdominal (visceral) obesity indicators. A study showed that the WHR was indicated to be superior to BMI and WaC in the cardiovascular risk assessment [24]. In other study in 122 patients with chronic kidney disease, a strong correlation was reported between WaC and visceral adiposity [14]. HC is an indicator of gluteo-femoral adiposity, which is more common in women and less hazardous for health compared to visceral adiposity [25].

In the present study, mean body weight, BMI, WaC and HC values increased significantly in both treatment groups starting from 3 months post-transplant. WHR increased significantly in the first three months in the CsA group and the first six months in the Tac group compared to the baseline values but did not change significantly in the following months. WaC measurements in the early post-operative period may be misleading due to post-operative oedema in the abdomen and waist regions, especially in the skin and subcutaneous areas. Thus, WHR is overestimated in the first three-six months after surgery. Gradual improvement in oedema and increase in adiposity over the following months may increase WaC and HC, although there is no change in WHR. In our cohort, median BF% values in the pre-operative period were 22.8% in the CsA group and 23.0% in the Tac group. The BF% increased significantly after three months in the CsA group and 24 months in the Tac group up to the 48-month post-transplant period. Our findings provide little explanation for the differences between treatment groups regarding BF% change after transplantation since there is insufficient data about our patient’s dietary habits and lifestyle changes during the post-transplant period. In a past study conducted with transplant patients, glucocorticoid and Tac treatment were reported to be associated with increased BF% and post-transplant diabetes mellitus [26].

Basal body weight, BMI, WaC, WHR and BF% values of both groups were similar, and there was no difference between percentage changes in body weight, BMI, WaC, WHR and BF% throughout the study. The increase in mean HC values in the CsA group was significantly higher than in the Tac group (median 2.80% vs. 0.98% in the 1^st^ month, 7.84% vs 4.96% in the 12^th^ month, and 8.10% vs. 3.00% in the 24^th^ month, respectively). Although the increase in HC values in the 36^th^ (median 8.69% vs. 5.18%) and 48^th^ (median 8.13% vs. 5.24%) months was higher in the CsA group, the difference did not reach statistical significance. Both CNIs increased BMI and overall adiposity. Since the increase in WHR was similar in both groups, we can conclude that the higher HC increase in the CsA group compared to the Tac group did not affect the metabolic parameters. The earlier increase of BF% in the CsA group indicates that CsA increased overall adiposity, and the accumulation was predominantly in the femoral region. On the other hand, general body adiposity in the Tac group increased in a later period.

Our previous retrospective study showed that CsA use was one of the independent predictors of weight gain 12 months post-transplant [27]. Weight gain may be associated with the continued increase in adipose tissue due to hyperlipidemia and higher water-sodium retention after CsA treatment, alterations in steroid treatment dose and duration, and poor patient compliance with dietary recommendations [28, 29]. Our present study’s anthropometric assessment included WrC and NC measurements. WrC values showed a significant early increase at 3 and 6 months in the Tac group compared to baseline values but decreased significantly at 48 months in the CsA and Tac groups. Compared to baseline values, NC increased significantly between the 1^st^ and 24^th^ months in the CsA group and between the 1^st^ and 12^th^ months in the Tac group but did not change in the following months. No significant difference was observed between WrC and NC percentage changes in the CsA and Tac groups. The early increase in WrC in the Tac group may indicate that Tac-related weight gain results in more homogeneous adiposity. In recent years, wrist-ankle measurement has been used to determine cardiovascular risk factors such as insulin resistance and hypertension [30–32]. WrC measurement can give information about body bone structure and peripheral fat distribution. Insulin shows anabolic effects by binding to insulin-like growth factor-1 (IGF-1) receptors in osteoblasts and can change bone mass and density [33, 34]. In the pre-transplant period, many factors, especially uremia, deterioration in calcium-parathormone levels and a diet poor in protein, cause insulin resistance [35]. Glucocorticoids used after transplantation cause dose-dependent peripheral and central insulin resistance and suppress insulin-related lipolysis. They also cause less accumulation of adipose tissue in peripheral areas compared to the central visceral adipose tissue of the body. During the follow-up period, insulin sensitivity increases, insulin resistance decreases with decreasing doses of drugs, increasing urea elimination and improving calcium-parathormone levels. There was no significant difference between the percentage changes in NC values ​​in the two groups after transplantation. Also, concomitant steroid therapy rather than CNIs may be responsible for the increase in NC. As seen in patients with Cushing’s syndrome, increased NC values ​​may be associated with hypercortisolemia and trunk obesity due to decreased insulin sensitivity.

Dyslipidemia is common among kidney transplant recipients, and new-onset or worsening dyslipidemia has been associated with the use of sirolimus, CNIs (especially cyclosporine), and glucocorticoids. Increases in total- and LDL-cholesterol levels are more common [36]. CsA specifically causes an increase in very low-density lipoprotein (VLDL) cholesterol, LDL-cholesterol and triglyceride levels by inhibiting LDL-cholesterol receptor synthesis and lipoprotein lipase activity and increasing apolipoprotein C-III and proprotein convertase subtilisin/kexin type 9 levels [37]. Compared with pre-transplant values in our study, there was a significant increase in total-cholesterol and HDL-cholesterol values in both groups. Compared with pre-transplant values, there was an increase in LDL-cholesterol values up to the 24^th^ month in the CsA group and all months (except the 12^th^ month) in the Tac group. No significant change was observed in triglyceride values. In our study, the changes in the percentage of total cholesterol were significantly higher in the CsA group in the 1^st^ and 12^th^ months than in the Tac group, suggesting that the hyperlipidemic effect of CsA was more prominent in the first year. Percentage changes in other lipid parameters of the groups were also comparable. In our study, the proportion of patients with post-transplant dyslipidemia in the CsA group was significantly higher than in the Tac group. Increases in total-cholesterol, LDL-cholesterol and triglycerides appear to correlate with anthropometric measurement increases during both CsA and Tac treatments. Therefore, especially the hyperlipidemic effect of CsA may be related to adiposity.

The increase in WaC and insulin resistance is expected to lead to an increase in TG levels and a decrease in HDL-cholesterol levels. HDL-cholesterol levels increased in both groups, whereas both CNIs did not change TG levels. This status can be explained by reducing post-transplant insulin resistance in uremic patients. However, our study did not measure insulin resistance. LDL cholesterol is the lipid fraction most closely associated with atherogenicity. Increasing WHR and WaC values ​​may increase atherogenicity and thus LDL-cholesterol levels. Many factors may have affected our study's analysis results regarding lipid parameters. During follow-up, physicians may have reduced doses or discontinued the drug in some patients using statins, mainly due to concerns about its interaction with CsA and graft dysfunction due to the risk of myopathy. An antilipidemic drug may be added to the treatment afterwards. In addition, these patients use many drugs that may affect insulin resistance.

In our study, Tac was not preferred in diabetic patients due to its higher diabetogenic potential than CsA, especially if the recipient has low immunological risk. Pre-transplant diabetes ratios were 18.3% in the CsA group and 1.5% in the Tac group. The difference in diabetic ratios of the groups may explain the higher basal serum glucose levels in the CsA group compared to the Tac group. When compared with baseline values, serum glucose levels decreased in the 12^th^ months in the CsA group and increased in the 1^st^ month in the Tac group. There were no significant changes at other time points. On the other hand, glucose percentage changes at 6 (median 5.40% vs. –7.78%, *p* = 0.049), 12 (4.41% vs. –7.76%, *p* = 0.030) and 24 (4.72% vs. –4.47%, *p* = 0.045) months were significantly higher in the Tac group than in the CsA group, respectively. CsA and Tac increase the risk of post-transplant diabetes [38]. However, Tac is more diabetogenic than CsA because Tac causes more severe swelling and vacuolization of islet cells [39]. Both drugs cause reversible toxicity in pancreatic beta cells, especially in the early post-transplantation period, directly affect the transcriptional regulation of insulin expression, cause glucose intolerance and inhibit lipolysis [40–44].

The relatively higher percentage of diabetes patients in the CsA group may also have been a factor in weight gain. However, the less diabetogenic effect of CsA in these patients, the use of lower doses of steroids and faster dose reduction for glycemic control may have facilitated glycemic control. In diabetic patients in the CsA group, impaired insulin-glucose homeostasis leads to hypertrophy of adipocytes and fat accumulation, especially in the lower part of the body [40, 45]. Tac and CsA can inhibit glucose entry into the cell by increasing the internalization of glucose transporter-4 (GLUT4) at different rates on the cell surface of adipocytes and by causing phosphorylations on insulin receptors at various points.

Post-transplant diabetes mellitus is a multifactorial condition that occurs in 4–25% of kidney transplant patients and within the first three months of transplantation in most cases [46, 47]. In a meta-analysis of three large-scale studies including 980 transplant patients, Tac therapy was associated with a 5.03-fold higher risk of post-transplant diabetes mellitus development [48]. However, post-transplant diabetes developed in more patients in the Tac group (19.1%) than in the CsA group (8.3%) during follow-up. After 36 months, weight gain in the whole cohort tended to increase more rapidly in the Tac group, which may support the continued diabetogenic effect of Tac in the late period. Although patients often received a similar corticosteroid regimen, we administered different doses to some patients. The steroid dose they were exposed to could have been very important in anthropometric measurements. However, we did not detect any difference in cumulative steroid doses between the groups in all months throughout our study. Depending on the use of CNIs at different doses and durations, fat distribution may also differ due to its diverse effects on glucose uptake [49, 50]. In our study, heterogeneous distribution of diabetic patients in the groups, insulin doses and cumulative steroid doses might have affected our results. Nephrologists preferred CsA or Tac treatment for some diabetic patients due to medical concerns. Transplantation is associated with an increased need for insulin. While most patients received post-transplant insulin therapy, very few patients used only oral antihyperglycemic drugs. Insulin has significant anabolic effects and could have affected study parameters, including weight gain. Even the insulin dose requirements were different during follow-up in patients with type 1 and type 2 diabetes and de novo diabetes mellitus.

Two subgroup analyses evaluated the effects of factors such as heterogeneity in fat distribution and the anabolic effect of insulin therapy on anthropometric measurements in diabetic and de novo diabetic patients. When compared anthropometric measurements in diabetic and non-diabetic patients, only baseline BF% values were significantly higher in patients with diabetes than in non-diabetics. The rates of increase in NC in the 12^th^ month in diabetic patients and BF% at the 24^th^ and 36^th^ months in non-diabetics were significantly higher than the other group. When anthropometric measurements in insulin-using and non-insulin-using patients were compared, the baseline anthropometric measurements were comparable. The increase rates in NC from the 3^rd^ month to the 24^th^ month and WaC in the 12^th^ month were significantly higher in patients who used insulin than in those who did not. Insulin treated patients are under the anabolic effect of the drug which can cause fat accumulation. Diabetogenic drugs given for the prevention of transplant rejection, mainly steroids, can cause fat accumulation in the central parts of the body like neck and waist. This accumulation increases insulin resistance further, requiring a higher dose to overcome poor glycemic control. This vicious circle can explain the difference between diabetic and non-diabetic subjects and insulin and other antihyperglycemic drug users among the diabetic group.

Metabolic disorders that can lead to hyperglycemia, insulin resistance, hypertension, dyslipidemia after kidney transplantation increase the risk of overweight and obesity [10]. A recent study revealed a significant association between greater weight gain and the youngest age, female gender, lower pre-transplant BMI, living kidney donor, and fewer post-transplant hospitalizations [51]. Another study showed that shorter pre-transplant dialysis time, a living kidney donor, and being obese at baseline increased the risk of weight gain [52]. Weight gain is expected in all recipients, especially in the early post-transplant period. In the present study, we determined obesity risk factors according to different anthropometric parameters beyond weight gain in a more extended period (4-year follow-up). We investigated the factors that were the primary statistically determinant of the development of obesity according to the patient’s BMI, BF%, WaC and WHR values at the end of the 48^th^ month. These risk factors included baseline BMI, young age, and lack of exercise for BMI; baseline BMI, baseline WrC, eGFR level at one month, baseline NC, and no exercise for BF%; baseline WaC, BMI and WrC values for WaC and baseline WaC, history of dyslipidemia and no exercise for WHR. After adjusting for other factors, a high baseline BMI increased the risk of being obese (based on BMI, BF% or WaC) by 1.49–2.09 times. Lack of regular exercise increased the risk of obesity (based on BMI, BF% or WHR) by 4.75–30.93 times. High 1-month eGFR increased the risk of obesity 1.04-fold (based on BP) and high baseline NC 1.49-fold. High baseline WaC increased the risk of obesity based on WaC by 1.18 times and the risk based on WHR by 1.11 times. In those with a history of dyslipidemia, the probability of developing obesity based on WHR was 4.66 times higher than those without a history of dyslipidemia. A recipient with a high baseline BMI, NC and WaC may have a higher risk of developing obesity due to hyperplasia of adipose cells. Dyslipidemia and not regular exercise can also increase the risk of obesity by increasing insulin resistance or as an indicator of high insulin resistance. Good early graft function (a higher eGFR) may trigger an increase in appetite and weight gain due to the improvement of uremic symptoms and metabolic changes, possibly with the contribution of immunosuppressive drugs. Age increase was associated with a 10% reduction in the risk of obesity according to BMI. Kidney recipients paying more attention to their health or reducing their food intake in old age may be associated with a decrease in BMI. High baseline WrC was associated with a 70% and 50% reduction in obesity risk according to BF% and WaC, respectively. WrC with less adipose tissue accumulation may not be a predictor of obesity in these patients.

Nephrotoxicity is considered the most critical side effect of CNI treatment. CNIs lead to graft dysfunction via dose-dependent renal vasoconstriction and the development of tubular atrophy and interstitial fibrosis in the chronic course [53]. In the current study, while 6^th^ and 48^th^-month eGFR values were significantly lower than the 1^st^-month levels in the Tac group, no significant difference was noted between Tac and CsA groups in terms of serum creatinine and eGFR levels during the entire 4-year post-transplant period. Similarly, in a past study by Alghamdi et al. [54], in CsA and Tac-treated patients, no significant difference was reported between treatment groups regarding 2-year follow-up data on serum creatinine levels. In two studies with 5-year follow-up of CNI-treated patients by Kaplan et al. [55] and Vincenti et al. [56], the authors noted significantly higher eGFR in the Tac group. The decrease in eGFR values in our patients receiving Tac therapy within the 6^th^ month of transplantation could be explained by the CNI toxicity, urinary tract infection and inadequate oral intake. However, we did not detect any difference between the episodes of urinary infection in the two groups in the first year after transplantation. Implementation of serum urea and creatinine measurements only at a single time point is an essential limitation of the current study, given the likelihood of fluctuations in serum urea and creatinine levels between measurement dates as well as the changes in clinical condition with possible impact on the graft function such as the development of acute kidney injury.

The main limitations of this single-centre study are the relatively small number of patients, the inability to extend the study over a longer period, and the lack of detailed information about patients' dietary adherence, water consumption, and other medications patients take. One hundred twenty-eight patients were included in the study, and 111 completed the study after four years. The younger age of Tac patients, which may be related to the physicians’ preference of prescribing Tac in younger patients to avoid the remarkable cosmetic side effects of CsA therapy (i.e. hirsutism, gingival hypertrophy) in this age group, might have also affected the findings achieved in the current study.

## Conclusion

In conclusion, our study shows that weight gain steadily increases after a successful kidney transplant. Many factors such as changes in dietary habits, immunosuppressive medications and concomitant diseases play a role in weight gain. Especially abdominal obesity increases the risk of cardiovascular disease. Our study suggests that especially being obese before transplantation, not exercising regularly and the presence of dyslipidemia increase the probability of developing obesity. The present study found similar anthropometric changes with CsA and Tac treatment in kidney transplant recipients. However, regression analysis showed that CNI type was not a risk factor for the development of obesity. In long-term follow-up, the potential disadvantages of hyperlipidemic in CsA and diabetogenic in Tac should not be ignored. In conclusion, transplant physicians should also focus on approaches to reduce weight gain in these patients. Future large-scale prospective studies with longer follow-ups after transplantation are needed to understand better the relationship of immunosuppressive therapy with anthropometric changes and cardiovascular risk factors in transplant patients.

## Data Availability

Datasets generated and/or analyzed during the current study are available upon reasonable request. It is sufficient to contact the corresponding author of the study, Emel Isiktas Sayilar emelisiktas@yahoo.com, upon request.

## Abbreviations

BMI: Body mass index
CKD-EPI: Chronic Kidney Disease Epidemiology Collaboration
CNIs: Calcineurin inhibitors
CsA: Cyclosporine-A
DBP: Diastolic blood pressure
EC-MPS: Enteric-coated mycophenolate sodium
GFR: Glomerular filtration rate
GLUT4: Glucose transporter-4
HLD: High-density lipoprotein
IGF-1: Insulin-like growth factor-1
LDL: Low-density lipoprotein
MMF: Mycophenolate mofetil
SBP: Systolic blood pressure
Tac: Tacrolimus
